# Biochemical and molecular responses of *Rosa damascena* mill. cv. Kashan to salicylic acid under salinity stress

**DOI:** 10.1186/s12870-022-03754-y

**Published:** 2022-07-27

**Authors:** Mohammad Omidi, Azizollah Khandan-Mirkohi, Mohsen Kafi, Zabihollah Zamani, Ladan Ajdanian, Mehdi Babaei

**Affiliations:** 1 Department of Horticulture Science, College of Agriculture and Natural Resources, University of Tehran, Karaj, 31587 Iran; 2 Department of Horticultural Sciences, Faculty of Agriculture, Ferdowsi University of Mashhad, Mashhad, Iran

**Keywords:** Antioxidant, Ion leakage, *Rosa damascena*, Salinity stress, Salicylic acid

## Abstract

**Background:**

Today, salinity stress is one of the most important abiotic stresses in the world, because it causes damage to many agricultural products and reduces their yields. Oxidative stress causes tissue damages in plants, which occurs with the production of reactive oxygen species (ROS) when plants are exposed to environmental stresses such as salinity. Today, it is recommended to use compounds that increase the resistance of plants to environmental stresses and improve plant metabolic activities. Salicylic acid (SA), as an intracellular and extracellular regulator of the plant response, is known as one of these effective compounds. Damask rose (*Rosa damascena* Mill.) is a medicinal plant from the Rosaceae, and its essential oils and aromatic compounds are used widely in the cosmetic and food industries in the world. Therefore, considering the importance of this plant from both medicinal and ornamental aspects, for the first time, we investigated one of the native cultivars of Iran (Kashan). Since one of the most important problems in Damask rose cultivation is the occurrence of salinity stress, for the first time, we investigated the interaction of several levels of NaCl salinity (0, 4, 8, and 12 ds m^− 1^) with SA (0, 0.5, 1, and 2 mM) as a stress reducer.

**Results:**

Since salinity stress reduces plant growth and yield, in this experiment, the results showed that the increase in NaCl concentration caused a gradual decrease in photosynthetic and morphological parameters and an increase in ion leakage. Also, increasing the level of salinity stress up to 12 ds m^− 1^ affected the amount of chlorophyll, root length and leaf total area, all of which reduced significantly compared to plants under no stress. However, many studies have highlighted the application of compounds that reduce the negative effects of stress and increase plant resistance and tolerance against stresses. In this study, the application of SA even at low concentration (0.5 mM) could neutralize the negative effects of salinity stress in the *Rosa damascena*. In this regard, the results showed that salinity increases the activity of antioxidant enzymes catalase (CAT) and superoxide dismutase (SOD) and the concentration of proline, protein and glycine betaine (GB). Overexpression of antioxidant genes (Ascorbate Peroxidase (APX), CAT, Peroxidase (POD), Fe-SOD and Cu-SOD) showed an important role in salt tolerance in Damascus rose. In addition, 0.5 mm SA increased the activity of enzymatic and non-enzymatic systems and increased salinity tolerance.

**Conclusions:**

The change in weather conditions due to global warming and increased dryness contributes to the salinization of the earth’s surface soils. Therefore, it is of particular importance to measure the threshold of tolerance of roses to salinity stress and the effect of stress-reducing substances in plants. In this context, SA has various roles such as increasing the content of pigments, preventing ethylene biosynthesis, increasing growth, and activating genes involved in stress, which modifies the negative effects of salinity stress. Also, according to the results of this research, even in the concentration of low values, positive results can be obtained from SA, so it can be recommended as a relatively cheap and available material to improve production in saline lands.

## Introduction

One of the most important ornamental plants in the world market is the rose, this plant is generally sold as cut, potted, and landscape flowers. In addition, rose petals are a natural source of fragrance and essential oils. Rose petal oil, called Rosatom is used as a valuable product in the perfume industry [1]. Due to the widespread drought conditions of recent years and the lack of confident water resources, saline soil levels are increasing, especially in arid regions. Given the background of Damask rose cultivation in dry and saline areas [2] and considering the potential of this plant to withstand environmental stresses, this plant seems to be an effective option for developing cultivation in these areas. The most important consequence of drought stress in soils is salinity, which occurs as a result of excessive solute accumulation, and the predominant mineral in saline soil is NaCl. In addition, the accumulation of minerals in soils increases due to the low precipitation [3]. Plant responses to salinity involve various changes in the activity of genes and proteins that consistently lead to changes in plant metabolism. These pathways often diverge from the primary metabolic pathways by the transcription of the primary gene [4]. Antioxidant defensive systems in plants that act enzymatically or non-enzymatically play a special role in reducing or inhibiting various environmental stresses. Enzymatic antioxidants include super oxide dismutase (SOD), CAT, POD, glutathione reductase (GR), and APX, which accumulate in higher plants under salinity stress to avoid oxidative damage. Among various types of antioxidant enzymes, SOD, CAT, GR, APX, and POD can be mentioned, which accumulate in plants under salt stress to avoid oxidative damage [5, 6]. Increase in APX activity under salinity and water stress has been reported in plants [7]. Today, the use of compounds that increase plant resistance to environmental stresses are recommended. One of these compounds is SA [8]. This compound is a growth regulator that, as a phenolic compound in plants, plays an essential role in regulating plant physiological processes, protecting plants against environmental stress and improving plant growth [9, 10]. SA ameliorates the negative effects of salinity stress by increasing pigment content [11], preventing ethylene biosynthesis [12], and increasing growth [13]. Researchers have concluded that the exogenous use of SA at low concentrations (1.0 mM) increases the effectiveness of antioxidants in the plant [14–16]. However, treatment with SA has been shown to temporarily reduce CAT activity and increase H_2_O_2_ levels, possibly playing a key role in systemic acquired resistance and resistance to oxidative stress [17]. Exogenous application of SA on to tomato plants has been shown to increase the activity of antioxidant enzymes such as CAT, POD, APX, and SOD, as well as proline content [9]. SA treatment has been reported to cause the accumulation of abscisic acid. Because abscisic acid synthesis stimulates a wide range of antioxidant proteins, it increases the plant’s resistance to stress. These results suggest that the activity of antioxidant enzymes is directly or indirectly regulated by SA and protects the plant from environmental stresses [18, 19]. Damask rose was found to be somewhat tolerant to salinity but its exact tolerance has not been determined. Therefore, the aim of the present study for the first time is to determine the morphological and physiological responses of Damask rose cv. Kashan associated with enhanced tolerance to salinity stress resulting from the application of SA and to study the effect of SA on antioxidant enzyme mechanisms.

## Material and methods

Uniform *Rosa damascena* cv. Kashan plants, propagated by tissue culture, were used as plant materials. Plants were cultivated in 10-L pots containing a combination of loamy-clay field soil, sand, and leaf compost (1:2:4, as volume base ratios, respectively) in Department of Horticulture science, University of Tehran. The average of field capacity (by weight) of pots was 90%. The pots were fertilized every two weeks with 20:20:20 fertilizer to prevent nutrient deficiency and over irrigated once every two weeks for leaching to prevent salt excessive accumulation. First, the concentrations of the main elements as well as EC and pH of the soil and the quality of irrigation water were evaluated (Tables 1 and 2). The mean maximum/minimum temperatures during the experiment were 31/12 °C and the relative humidity ranged between 30 and 64%. The average solar radiation was 10,132 (Rad. T.S.R. (kj m^2^)). Different levels of NaCl salinity including 0 (control), 4, 8, and 12 ds m^− 1^ and SA levels of 0, 0.5, 1, and 2 mM were used as treatments for 45 days when leaves fully expanded in spring. SA was first dissolved in ethanol and then diluted to the considered volume. SA treatments were performed by two sessions of foliar spray (100 mL/plant), one just before starting salinity stress treatment and the other three weeks after the start of salinity stress.

**Table 1:** Physical and chemical characteristics of potted soil (field soil, sand and leaf compost as 4:2:1 of volume base ratios, respectively) for cultivation of Damask rose cv. Kashan

Traits	Value
pH	6.52
EC (ds m^− 1^)	0.162
N (%)	1.1
P (ppm)	31.2
K (ppm)	38.74
Texture	Loam-clay

**Table 2:** Chemical characteristics of water used in this study

Chemical characteristic	Value
pH	6.86
EC (ds m^− 1^)	0.9
Cl (mg L^− 1^)	92.6
HCO3^−^	86.2
Ca (ppm)	230
Mg (ppm)	43
Na (ppm)	68

### Morphological characteristics

At the end of the experiment, plants were harvested and the substrate was washed from the roots. The leaf area (LA, cm^2^ plant) was measured (using Delta-T Devices Ltd., Cambridge, UK) in all plants. Plant height, root length, leaf and shoot fresh and dry weights (DW) and root fresh and dry weights per plant were determined, after drying in the oven at 70 °C for 48 h to reach constant weight in all plant materials [20].

### Relative water content (RWC)

Fresh leaf and stem samples were weighted to determine fresh weight (FW), and then they were immersed in distilled water at 4 °C for 24 h. After absorption of surface water, they were weighed again to determine the turgid weight (TW). Leaf samples were then dried in the oven at 75 °C for 48 h and their DW was measured. Values of FW, TW, and DW were used to calculate leaf relative water content (LRWC) using the following equation [21].

$$RWC\ \left(\%\right)=\left[\left( FW- DW\right)/\left( TW- DW\right)\right]\times 100$$

### Electrolyte leakage of leaves

Ten leaves were taken randomly from each plant, thoroughly washed with distilled water, and placed in 50 mL falcons, into which 20 mL of distilled water was added. Then, the flasks were placed on the shaker for 24 h at room temperature, and their electrical conductivity (EC1) was measured. Samples were autoclaved for 10 min, and after cooling to room temperature, the electrical conductivity (EC2) was measured. Electrolyte leakage is calculated according to the following equation as membrane damage [22].

$$EL\%= EC1/ EC2\times 100$$

### Determination of photosynthetic pigments

To determine the chlorophyll *a* and *b*, the youngest, fully expanded leaves of each plant were used. The leaves were extracted with acetone, and the concentrations of chlorophyll *a* and chlorophyll *b* were determined by Plate reader (EON, Bio Tek America) at 645 nm, 663 nm, respectively [23].

### Extraction for enzymatic measurements

0.5 G of leaf samples were weighed and powdered with liquid nitrogen and after adding 500 μL extraction buffer (0.15 mM Tris with pH = 7.5) and 50 mg of polyvinylpyrrolidone, they were crushed in a mortar, and then the samples were transferred to 1.5 mL flasks and centrifuged at 10000 rpm for 10 min at 4 °C. the supernatant was transferred to new falcons and was used to measure the enzymatic activities [24, 25]

### Enzymatic activities

#### Superoxide dismutase (SOD)

The activity of SOD was measured by determining the amount of enzyme that inhibited the rate of nitroblue tetrazolium (NBT) by 50% at 560 nm. The reaction mixture, consisting of 0.5 M PBS (pH 7.5), 0.1 mM EDTA, 13 mM methionine, 63 mM NBT, 1.3 mM riboflavin, and 0.1 mL enzyme extract, in 5.0 mL test tubes were illuminated for 15 min at 25 °C and using a non-illumination surface as blank. One unit of enzyme activity was defined as the quantity of SOD required to produce a 50% inhibition of reduction of NBT [5].

#### Catalase (CAT)

Catalase (CAT) enzyme activity was measured by the decrease of absorbance at 240 nm attributed to the decomposition of hydrogen peroxide (H_2_O_2_). The reaction mixture consisted of 2.6 mL of 50 mM phosphate buffer (pH 7) containing 0.4 mL of 15 mM H_2_O_2_, and 0.2 mL of the enzyme extract using spectrophotometer model Lambda EZ 201. The enzyme activity was expressed in units mg^− 1^ protein [24].

#### Guaiacol peroxidase (GPX)

The activity of guaiacol peroxidase (GPX) enzyme was measured by using reaction medium containing 50 mM phosphate buffer (pH 7), 9 mM guaiacol, and 19 mM H_2_O_2_. The enzyme activity was measured by monitoring the increase in absorbance at 470 nm during polymerization of guaiacol [25].

#### Ascorbate peroxidase (APX)

The activity of the APX enzyme was measured by the Agarwal et al., (2005) method [15]. The reaction mixture contained 550 μL of 50 mM phosphate buffer (pH 7.6), 100 μL of 1 mM EDTA, 100 μL of the extract, and 250 μL of 0.25 mM ascorbic acid. The reaction was monitored using a spectrophotometer (Lambda EZ 201) at 290 nm.

### Physiological measurements

#### Proline

Proline was measured according to the method of Bates et al. [26]. Leaves (0.5 g) were ground in 10 mL of 3% sulfosalicylic acid. The obtained extraction was centrifuged (napco 2028R, USA) for 5 min in 10,000 rpm, and then 1 mL of the upper liquid were mixed with 1 mL of acid ninhydrin solution and 1 mL of acetic acid and placed on a shaker for 20 minutes. The samples were then heated at 90 °C for 1 hour, cooled in ice water to stop all reactions, thoroughly mixed with 4 mL of toluene, and incubated 20 min at room temperature. After that, the supernatant was used for the measurement of absorbance at 520 nm. The proline concentration was determined using a calibration curve for proline as μM g^− 1^ FW.

#### Glycine betaine (GB)

GB was determined in leaves following the method of Grieve and Grattan (1983). Leaf samples (0.5 g) were powdered and homogenized in 25 mL of distilled water, then mechanically shaken for 48 h at 25 °C. The extract (1 mL) was mixed with 1 mL of 1 N H_2_SO_4_ and 0.4 mL of potassium tri-iodide solution and was kept in 4 °C for 24 h followed by centrifugation at 15,000 g for 15 min. The supernatant was carefully extracted with a fine-tipped glass tube, and the crystal sediments were dissolved in 9 mL of 1, 2-dichloroethane. After 2 h, samples were measured with a spectrophotometer at 365 nm (Lambda EZ 201). The concentration of GB was calculated using the standard curve, and the results were expressed as μmol g^− 1^ FW [27].

#### Malondialdehyde (MDA)

0.5 g of leaf was homogenized in 5 mL of 0.1% mL trichloroacetic acid (TCA) and then centrifuged at 10,000 rpm. 2 mL of this solution and 2 mL of 0.5% thiobarbituric acid (TBA) were boiled in 95 °C boiling water bath for 30 min and then quickly cooled in an ice bath. After centrifugation at 10,000 g for 10 min, the absorbance of the supernatant was recorded at 532 and 600 nm. The measurements made at 600 nm were deduced from those at 532 nm, and the levels of MDA was expressed as nmol g^− 1^ FW [28].

#### Total phenolics (TP)

Total phenolic content (TPC) of leaf samples was measured by Folin–Ciocalteu method [29] with some modifications using a plate reader (EON, Bio Tek America) at 725 nm. For this measurement, 0.5 g of the fresh samples of leaf were homogenized in 1.5 mL of 80% methanol and centrifuged at 15,000 rpm for 15 min. Then, 10 μL of supernatant was removed using a sampler and added into the plate well, then 75 μL of 10% Folin–Ciocalteu was added to the well, then 75 μL of 6% sodium carbonate was added to the reaction mixture. The measurement was compared to the standard curve of gallic acid solution and expressed in mg gallic acid g^− 1^ FW.

### Antioxidant capacity measurement

The antioxidant capacity (AC) of the extracts was determined by free radical neutralization (DPPH) (2 and 2 diphenyl-1-pyridyl hydrazine). 0.5 g of fruit tissue was homogenized in 4 mL of 80% methanol. The mixture of fruit tissue and ethanol was centrifuged at 9500 rpm for 20 min. Then, 3.4 mL of 60 μ mol solution DPPH was added to 100 μL methanol extract. After incubation, the absorbance of samples was determined at 517 nm using a microplate reader (EON, Bio Tek America) [30].

### Gene expression analysis

After applying salt stress (45 days from the start of stress) on the plants, sampling was done from fully developed and young leaves. The samples were then stored at − 80 °C until RNA extraction. RNA extraction was carried out using the Invisorb® Spin Plant RNA Kit (Invitek Co.).

### Determining quality and quantity of RNA, cDNA construction, and designing primer

To determine the quality and quantity of the extracted RNA, spectrophotometry method at 260 and 280 nm using NanoDrop (NanoDrop 2000C) and electrophoresis agarose gel were used. To remove possible DNA contamination in the extracted RNA, RNA treatment with DNAse1 was performed based on the protocol and using a Thermo Scientific Kit. Thermo Scientific Kit was used to convert RNA to cDNA. The primers were designed according to the results of the genome of *Rosa chinensis* [31, 32] and using clone manager and Notepad ++ software and were arranged via Eurofins Genomics (Table 3).

**Table 3:** Sequence of primers used for real-time qRT-PCR in Damask rose

Gene name (MDP ID)	Abbreviation	Primer sequence (5′–3′)	Fragment Length (bp)	Amplification efficiency (%)	Coefficient of Determination R^2^
SAND family protein RC5G0507300	SAND	Forward Primer: GTGGAGGTGGTGGTATTCTG Reverse Primer: CTAACTGCCGCAATCTCATC	151	91.3	0.999
protein phosphatase 2A	PP2A	Forward Primer: AACTGTCGAACCAGCTCATC Reverse Primer: ATTTGTCCTGCGAGAAGTTG	185	95.5	0.992
Elongation factor 1-alpha RC5G0521000	EF1	Forward Primer: TGAAGCTGGTATCTCCAAGG Reverse Primer: GGTGGCATCCATCTTGTTAC	106	92.5	0.999
Tubulin alpha-2 chain	TUB	Forward Primer: GTTGGTGGTGGTACAGGTTC Reverse Primer: TTCAAGGAGGGCATGAGTAG	168	93.8	0.999
Ascorbate peroxidase RC5G0530600	APX	Forward Primer: GGTTACTGGTGGACCTGATG Reverse Primer: GTGGTCAGAACCCTTGACAG	103	90.1	0.999
Catalase RC7G0342400	CAT	Forward Primer: CCATTATTGTCCCTGGTGTC Reverse Primer: TGTTGTGGTGAGCATTCTTG	144	92.3	0.998
Peroxidase RC5G0029500	POD	Forward Primer: GCAGAGCAGAGATCCTTGAG Reverse Primer: TTCAGAGCTGGGTCAACTTC	190	91.3	0.998
Fe- Superoxide dismutase RC4G0497500	FeSOD	Forward Primer: CTGTTGCTTGAAATGCTTTG Reverse Primer: AGGGCAACTCAGCACAATAG	171	91.7	0.990
Cu- Superoxide dismutase RC3G0373000	CuSOD	Forward Primer: GAGATGGCCCAACTACTGTG Reverse Primer: AGCAGGATTGAAGTGTGGTC	128	94.5	0.999

### Relative gene expression

To confirm the quality of cDNAs and primers, a reaction mixture was made using EF1-a, UBC, and ACT reference gene primers on the constructed cDNAs, and the gene expression was measured by the qPCR instrument (BioRAD,USA). The reaction mixture was made based on the information in Table 4 and performed according to the specifications of the qPCR reaction. The Real-Time PCR reaction was done by the 5x HOT FIRepol Kit, based on Siber Green qPCR Mix Plus. The thermal profile was defined based on the device protocol. Gene expression was estimated by the method of efficiency adjusted ∆∆CT (Table 4).

**Table 4:** Real Time PCR reaction mixture

Compound	Amount (μL)
cDNA (1:10 diluted)	**6**
Siber Green qPCR Mix	**4**
Forward primer (10 μM)	**5**
Reverse primer (10 μM)	**5**
DEPC water	**10**
Total volume	**30**

### Statistical analysis

The experiment was performed as a factorial trial with a randomized complete block design with 4, 8 and 12 ds m^− 1^ salinity treatments and irrigation water as a control (0.9 ds m^− 1^) as control along with 0, 1, 1.5- and 2-mM SA treatments and control without SA in four replications and three experimental units in each replication. For analysis of variance (ANOVA), SAS 9.1.3 and SPSS 19.0 software were used and mean comparison was done based on Tukey’s Test at 1 and 5% probability level.

## Results and discussion

### Plant growth and development

The fresh and dry weights of the leaves of the Damask rose (*Rosa damascena*) were significantly affected by the application of SA and salinity stress. According to the results (Table 5), non-application of salinity stress and the application of SA at levels of 0.5 mM involved the highest fresh (2.99 g) and DW (1.49 g) of the leaves, respectively. In addition, the highest level of salinity treatment (12 ds m^− 1^) and non-application of SA reduced the fresh weight of the leaf by 49% (Table 5); similarly, the lowest amount of leaf dry weight (0.97 g) was observed in the aforementioned treatment (Table 5). Therefore, it can be concluded that SA can play an effective role in raising the plant’s salt tolerance and protecting it from damages caused by salinity. Regarding the weight of the aerial organ, results demonstrated that the fresh weight reduced by 57% (Table 5) after increasing the salinity level (Treatment 12 ds m^− 1^) and applying SA at a concentration of 2 mM; meanwhile, the control treatment (non-application of salinity stress) involving SA 1 mM resulted in the highest fresh weight of the aerial organ (275.76 g) (Table 5). Moreover, the dry weight of the aerial organ was affected by the salinity stress and reduced by 42% at the salinity level of 12 ds m^− 1^, compared to treatments with no stress (Table 5). Also, no significant differences were observed between levels 8 and 12 ds m^− 1^; consequently, it can be concluded that plants reduce their dry weight as a response to salinity, at the onset of the stress up to a specific level. However, as the salinity stress increases, osmolytes accumulate and the dry weight increases as well. The highest fresh weight of the root was found to be 176.1 g, after the application of SA at 1 mM concentration and the non-application of salinity stress; this value reduced by 71% under level 12 ds m^− 1^ salinity and SA at a concentration of 0 (Table 5). Additionally, based on the obtained results, the highest and lowest DW of the root were found to be 17.52 g and 54.84 g, in salinity levels of 12 and 8 ds m^− 1^, respectively (Table 5). Thus, no significant differences were observed between level 4 ds m^− 1^ and absence of stress; subsequently, it can be concluded that the salinity stress reduced the fresh weight of the root in *Rosa damascena*, yet the reduction continued until level 8 ds m^− 1^ which was found to be the case for the dry weight as well. On the other hand, the application of SA decreased the effects of salinity stress, therefore increasing the fresh weight of the root at different levels of salinity to a considerable extent. Given the comparison between the mean values, the best level of SA was found to be 1 mM at all salinity levels.

**Table 5:** Mean comparisons for growth parameters of *R. damascena* plants under different salinity levels and treated with salicylic acid at four rates of application

salinity Treatment	SA	Leave		Shoot		Root		Root Length (cm)	plant height (cm)	leaf area (cm^2^)
		FW (gr)	DW (gr)	FW (gr)	DW (gr)	FW (gr)	DW (gr)			
Control	control	2.63 ± 0.35ab	1.34 ± 0.10ab	254.91 ± 7.29ab	165.10 ± 8.08ab	125.17 ± 5.59abc	56.11 ± 4.17ab	57.75 ± 7.00bc	86.33 ± 8.95ab	187.16 ± 11.89a
	0.5 mM	2.99 ± 0.39a	1.49 ± 0.16a	218.65 ± 5.07abc	188.55 ± 6.86ab	146.61 ± 11.29ab	54.76 ± 5.69ab	59 ± 3.38bc	99.32 ± 1.45a	174.89 ± 7.06ab
	1 mM	2.81 ± 0.40ab	1.41 ± 0.19a	275.76 ± 9.73a	200.19 ± 3.50a	176.1 ± 4.23a	70.66 ± 6.25a	76.25 ± 2.89a	91 ± 4.50ab	189.38 ± 5.03a
	2 mM	2.94 ± 0.35ab	1.32 ± 0.09ab	258.09 ± 7.48ab	184.97 ± 4.64ab	138.07 ± 11.48abc	67.75 ± 5.54ab	65.5 ± 4.78ab	80.66 ± 4.80ab	176.11 ± 5.15ab
4 ds m^−1^	control	2.82 ± 0.41ab	1.22 ± 0.14ab	226.31 ± 4.92abc	168.12 ± 6.38ab	131.83 ± 4.34abc	54.22 ± 3.35ab	63.75 ± 6.67ab	80 ± 11.53ab	164.66 ± 10.12abc
	0.5 mM	2.95 ± 0.38ab	1.24 ± 0.16ab	237.28 ± 4.81ab	166.75 ± 3.36ab	131.55 ± 5.65abc	52.98 ± 8.15ab	59.62 ± 1.74bc	84.67 ± 1.76ab	177.63 ± 8.82ab
	1 mM	2.98 ± 0.23ab	1.22 ± 0.12ab	209.79 ± 8.17abc	146.41 ± 3.94abc	154.18 ± 8.35ab	69.63 ± 6.62a	74.75 ± 4.64ab	88.66 ± 4.16ab	167.14 ± 9.10abc
	2 mM	2.93 ± 0.35ab	1.28 ± 0.15ab	262.94 ± 14.06ab	141.96 ± 8.70abc	140.09 ± 7.40ab	67.19 ± 8.53ab	59 ± 6.20bc	99.03 ± 1.85a	140.34 ± 7.55abc
8 ds m^−1^	control	2.38 ± 0.15b	0.98 ± 0.06b	162.81 ± 13.72bcd	162.31 ± 4.91abc	114.1 ± 4.37bcd	53.93 ± 6.30ab	70.75 ± 5.20ab	92 ± 6.65ab	144.20 ± 4.98abc
	0.5 mM	2.49 ± 0.18ab	0.98 ± 0.08b	178.14 ± 10.19bcd	107.05 ± 1.26bc	117.71 ± 10.39bc	46.87 ± 6.47ab	68.5 ± 5.47ab	99.33 ± 2.33a	130.86 ± 7.32bc
	1 mM	2.85 ± 0.18ab	1.05 ± 0.09ab	194.16 ± 13.77bcd	131.4 ± 5.40abc	134.82 ± 6.06bc	57.02 ± 2.90ab	67.75 ± 3.68ab	77.66 ± 3.92ab	135.03 ± 5.15bc
	2 mM	2.81 ± 0.24ab	1.18 ± 0.14ab	175.68 ± 8.85bcd	116.04 ± 2.26bc	125.16 ± 14.24bc	52.03 ± 2.11ab	54.25 ± 3.56bcd	84.66 ± 8.08ab	134.47 ± 10.47bc
12 ds m^−1^	control	2.26 ± 0.21b	0.97 ± 0.05b	210.58 ± 11.80abc	163.29 ± 4.68abc	101.17 ± 13.95d	52.68 ± 3.71ab	52.25 ± 3.47bcd	75 ± 11.53b	121.49 ± 9.12c
	0.5 mM	2.84 ± 0.42ab	1.15 ± 0.16ab	220.37 ± 13.08abc	163.15 ± 7.12abc	135.68 ± 15.19bc	52.41 ± 4.03ab	50 ± 2.79d	83.66 ± 3.66ab	122.23 ± 4.42c
	1 mM	2.85 ± 0.20ab	1.11 ± 0.06ab	199.68 ± 8.22bcd	148.89 ± 4.04abc	127.26 ± 15.72bcd	68.97 ± 1.38ab	61 ± 6.98bc	93.66 ± 2.96a	131.60 ± 2.53bc
	2 mM	2.79 ± 0.31ab	1.12 ± 0.11ab	127.59 ± 10.54d	96.68 ± 6.78c	102.33 ± 10.22d	44.69 ± 1.02b	55.25 ± 8.50bcd	88.66 ± 4.66ab	139.44 ± 12.00abc

*DW* dry weight, *FW* fresh weight

The different letters within each column indicate significant differences according to Duncan’s multiple-range test (*P* = 0.05)

The results of examinations conducted on the root length trait showed that it was significantly affected by the application of SA and salinity stress. Accordingly, after increasing the level of salinity stress to 12 ds m^− 1^ and applying SA at 0.5 mM concentration, the lowest value of root length was observed (50 cm) (Table 5); however, this value increased by 34% after the application of SA at 1 mM concentration and non-application of salinity stress. As a result, increasing the salinity level reduced the root length, while the application of SA raised the depth of the root which, to a certain extent, can neutralize the negative effect of salinity. Based on this, the highest leaf area was observed in the treatment without salinity stress with salicylic acid at a concentration of 1 mM as 189.38 cm^2^, while the lowest leaf area (121.49 cm2) was observed in the salinity treatment of 12 ds m^− 1^ and no application of salicylic acid (Table 5).

The morphology of plants is initially affected by the consequences of salinity stress which depends on the severity and duration of stress as well as the type of the plant [33]. As the first consequence of salinity, reduction in leaf area decreases the potential photosynthesis ability of the plant. When exposed to salinity, plant cells become dehydrated and contracted; though the leaves would regain the lost volume after several hours, cell elongation would reduce [34]. After a few days, the reduced cell elongation and cell division would result in smaller leaves. Consequently, the leaves gradually age and become dried, and new leaf generation reduces. Finally, in case of continued exposure to salinity at high levels, plant death would occur. Salinity affects and prevents the division and elongation of plant growth zones either directly or indirectly. Branch growth is reduced due to salinity present in the growing tissues which does not form in mature photosynthetic tissues [34]. Rose, like other plants, is affected by stress, which affects its growth and development, with some changes in plant morphology depending on the stress levels [35]. In an experiment conducted to assess the salinity resistance of *Rosa chinensis*, salinity had a negative effect on shoot length, diameter, and DW [36]. The results of various studies have shown that the use of SA improves vegetative traits in plants under salinity stress [37–39]. The increase of root growth and maintenance of its health following SA treatment increases the absorption of water and nutrients, which ultimately leads to increased plant growth [40]. The increase in root growth has led to SA being recognized as an effective, efficient, and important phytohormone that increases root growth in important economic vegetables such as carrots, radishes, and beets [8]. SA increases the activity of rubisco enzyme, which improves photosynthesis and thus increases leaf area [41]. The concentration of 2 mM of SA in *Calendula officinalis* plants increased the leaf area with similar results for tomatoes, cucumbers, and strawberries [42].

### Relative water content (RWC)

Leaf relative water content (RWC) is one of the primary traits that is reduced significantly under salinity stress. Examinations in this study showed 41.08% decrease in leaf RWC under the highest stress level (12 ds m-1) and non-application of SA compared to the control condition where stress was absence and SA was applied at a concentration of 0.5 mM. Aerial organ RWC was also affected by the treatments; its highest amount was 65.75% which was observed in absence of salinity stress and application of SA at 0.5 mM concentration. Increasing SA concentration to 1 mM and applying salinity stress at the level of 12ds m-1 reduced the RWC to 57.17% (Table 6). Decreases in the RWC of plants under stress conditions may indicate the loss of turgor pressure, which results in limited water access to cells [43]. By affecting and disrupting membrane proton pumps, salinity reduces cell growth [35]. Under salinity stress, water absorption decreases with increasing salt in the root area, and therefore in such conditions the RWC decreases [44]. Ali et al. (2014) observed that salinity stress reduces the RWC of rose petals in the ‘trinity petal’ cultivar. In another study, salinity stress reduced leaf RWC in European wild rose species (*R. rubiginosa*) [45]. The use of SA improves the RWC in shoots of various plants [46, 47]. The positive effect of SA on the RWC under various stresses such as salinity in barley, corn, and wheat plants has been reported. Foliar application of SA increases the leaf RWC [13, 48, 49]. Therefore, SA increases RWC by maintaining cell turgor pressure, regulating the opening and closure of stomata, interacting with other plant growth regulators such as abscisic acid (ABA), and preventing water loss [50]. The results of the present study are consistent with the reports of the above researchers regarding the reduction of the RWC under salinity stress.

**Table 6:** Mean comparisons for chlorophyll content, RWC for leave and shoot and EC of *R. damascena* leaves under different salinity levels and treated with salicylic acid at four rates of application

salinity Treatment	SA	RWC (%)		EC of Leave (%)	Total Chlorophyll (mg g^− 1^.FW)	Chlorophyll a (mg g^− 1^.FW)	Chlorophyll b (mg g^− 1^.FW)
		leave	Shoot				
Control	control	75.27 ± 3.66ab	62.91 ± 8.83ab	54.60 ± 4.97ab	3.00 ± 0.61ab	1.69 ± 0.19abc	1.31 ± 0.32ab
	0.5 mM	87.85 ± 3.38a	65.75 ± 7.59a	41.34 ± 3.05c	3.02 ± 0.63ab	1.66 ± 0.20abc	1.35 ± 0.31ab
	1 mM	67.65 ± 2.14abc	48.19 ± 3.47abc	49.56 ± 1.67abc	2.70 ± 0.90ab	1.55 ± 0.38bc	1.14 ± 0.41ab
	2 mM	78.21 ± 3.73ab	47.86 ± 2.81abc	50.38 ± 1.32ab	3.86 ± 0.67a	2.42 ± 0.24a	1.43 ± 0.31a
4 ds m^−1^	control	81.41 ± 4.42a	46.65 ± 3.28abc	52.59 ± 4.15ab	2.92 ± 0.65ab	1.83 ± 0.29abc	1.08 ± 0.24ab
	0.5 mM	71.64 ± 3.54ab	50.16 ± 3.08abc	44.91 ± 2.94bc	2.80 ± 0.53ab	1.89 ± 0.26abc	0.90 ± 0.16ab
	1 mM	75.56 ± 2.27ab	52.07 ± 2.53ab	49.19 ± 5.19abc	3.56 ± 0.40ab	2.20 ± 0.10ab	1.35 ± 0.18ab
	2 mM	75.58 ± 3.05ab	58.98 ± 7.3abc	42.72 ± 4.45bc	3.22 ± 0.44ab	2.39 ± 0.10a	0.83 ± 0.23ab
8 ds m^−1^	control	66.45 ± 4.61abc	41.54 ± 3.55bc	60.11 ± 2.96a	2.40 ± 0.68b	1.65 ± 0.39abc	0.73 ± 0.17ab
	0.5 mM	54.62 ± 2.01c	48.99 ± 3.66 abc	50.11 ± 4.46ab	2.57 ± 0.80ab	1.74 ± 0.46abc	0.83 ± 0.22ab
	1 mM	73.97 ± 3.15ab	43.97 ± 3.92bc	54.29 ± 5.82ab	2.99 ± 0.72ab	2.07 ± 0.36ab	0.92 ± 0.24ab
	2 mM	59.68 ± 1.65bc	37.61 ± 2.51bc	57.64 ± 4.58ab	3.77 ± 0.62ab	2.37 ± 0.30ab	1.30 ± 0.22ab
12 ds m^−1^	control	51.86 ± 2.84c	34.96 ± 4.57bcd	60.90 ± 2.49a	2.39 ± 0.48b	1.47 ± 0.20c	0.62 ± 0.17b
	0.5 mM	68.04 ± 3.26abc	46.69 ± 2.66 abc	47.61 ± 1.73abc	2.45 ± 0.36ab	1.76 ± 0.14abc	0.69 ± 0.12ab
	1 mM	60.10 ± 2.08bc	28.16 ± 1.95d	48.85 ± 4.49abc	3.05 ± 0.35ab	2.14 ± 0.10ab	0.91 ± 0.14ab
	2 mM	61.21 ± 2.11bc	32.74 ± 1.2bcd	59.25 ± 4.22ab	3.69 ± 0.42ab	2.03 ± 0.18abc	1.06 ± 0.14ab

*EC* Electrical Conductivity

*RWC* Relative Water Content

*FW* Fresh Weight

The different letters within each column indicate significant differences according to Duncan’s multiple-range test (*P* = 0.05)

### Ionic leakage

Findings showed that leaf ion leakage is affected by the application of both salinity stress and SA. Differences were found between various levels of SA and salinity stress in terms of the extent of affecting ion leakage reduction. Accordingly, the application of salinity stress (12 ds m^− 1^) without SA involved the highest percentage of leaf ion leakage (60.9%) which reduced to 32.11% in plants that received SA at 0.5 mM concentration and remained unaffected by salinity stress (Table 6). Free radicals cause peroxidation of membrane lipids and the release of potassium ions from the cell wall. Sodium ions also replace potassium binding sites in cell membranes due to their competitive effect with potassium and because it cannot do potassium activity, it causes electrolytes to leak from the cell wall [51]. As the leaf relative water content decreases in salinity stress, the cells shrink and the cell membranes lose their stability [52]. Resulting in increased cell membrane permeability and the cell contents leak out of it [45]. The increase in membrane permeability in electrical conductivity stress is also associated with a decrease in calcium absorption and accumulation because calcium plays an essential role in maintaining membrane structure [53]. Numerous studies on roses have shown that ionic leakage also increases with increasing salinity [54]. The effectiveness of SA in reducing the ionic leakage in corn plants [13] and barley [46] were reported. By increasing the levels of polyamines of putresin, spermidine, and spermine and other cell-protecting compounds, SA increases and stabilizes the leaf membrane stability index and prevents ionic leakage by controlling membrane permeability [55]. Our results are consistent with the above findings.

### Chlorophyll content

The results of mean value comparison (Table 6) regarding the total chlorophyll content in the Damask rose leaves showed the highest amount of chlorophyll (the mean value of 3.86 mg g^− 1^ FW) in treatment samples involving no salinity stress and SA at 2 mM concentration level; however, the lowest chlorophyll content mean value was found to be 2.39 mg g^− 1^ FW under salinity stress (12ds m^− 1^) and non-application of SA treatment. It can be concluded that the application of SA increases the total chlorophyll contents of leaves and considerably compensates for the negative impact of salinity stress on chlorophyll reduction.

The highest content of chlorophyll *a* (2.42 mg g^− 1^ FW) was reported in treatment of 2 mM SA and there was no significant difference between other SA treatments (Table 6). The highest level of *chlorophyll b* (1.43 mg g^− 1^ FW) was found in the control treatment without salinity stress and its lowest level (0.682 mg g^− 1^ FW) in 12 ds m^− 1^. Evidently, increasing the level of salinity stress would decrease the amount of chlorophyll which could be due to the destruction of chlorophyll or leaf tissue and reduced photosynthesis. Increasing the level of salinity stress in this study also decreased the amount of chlorophyll; results related to the SA treatment showed that its application raises plant resistance and increases chlorophyll contents in the leaves of stressed plants. Salinity decreases non-stomatal factors such as RUBP carboxylase efficiency, Rubisco regeneration, mesophilic resistance and chlorophyll content [56]. Low SA concentrations (0.5–1 mM) increase the stomatal conductivity in plants under salinity stress [57]. Exogenous use of SA increases the photosynthetic rate, gas exchange, and stomatal conductance [58]. The use of SA increased the stomatal conductivity in soybeans and corn [37]. There have been some reports that the use of SA reduces the stomatal conductivity and closes the stomata, which has been linked to the association of SA with ABA, as well as its antiperspirant effects. These different effects of SA depend on its dosage [59].

### Total phenol content (TPC)

**Fig. 1:**
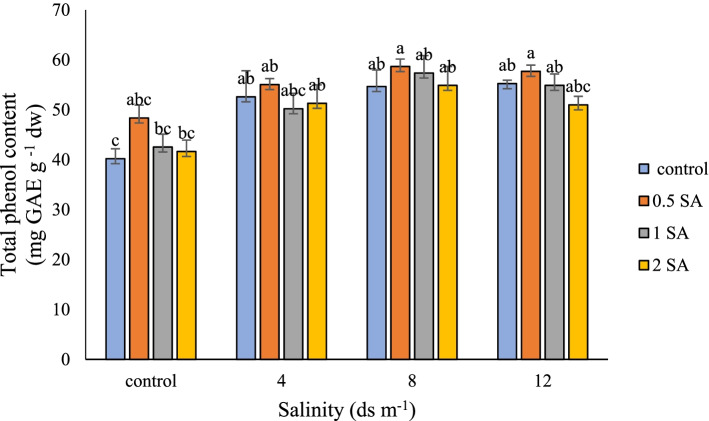
Comparison of the interaction of salinity and SA levels on the amount of total phenol in Damask rose. (*p* ≤ 0.01). Different letters indicate significant differences between treatments by Tukey’s Test.

Increasing the total phenolic content, which is considered as an antioxidant mechanism in plants, raises plant tolerance against stress. In this research, plants exposed to both stress (level 8 ds m^− 1^) and SA at 0.5 mM concentration contained the highest amount of total phenolic content (58.66 mg GAE g ^− 1^ DW); however, this value at its lowest was found to be 40.20 mg GAE g ^− 1^ DW in the control treatment (involving no stress or the application of SA) (Fig. 1). One of the non-enzymatic defense mechanisms for coping with oxidative stress induced by stress in plants is the accumulation of phenolic compounds. Plant phenols are secondary plant metabolites that are synthesized from the shikimic acid pathway and from phenylpropanoid metabolism under favorable environmental conditions, but different environmental stresses alter their content in cells [60]. When plants face salinity stress, the level of phenolic compounds increases, especially precursors of lignin biosynthesis, which leads to the thickening of the cell wall and creating a biological barrier for salt to enter the cells [61]. Due to the fact that SA is a phenolic compound, by increasing the production of non-enzymatic antioxidants such as phenolics, the plant resistance to oxidative stress increases [62]. Increased production of total phenolics and flavonoids has already been reported [61]. Increased production of phenolics by exposure to salinity stress confirm that they have a role in the alleviation of the destructive effects.

### Proline content

**Fig. 2:**
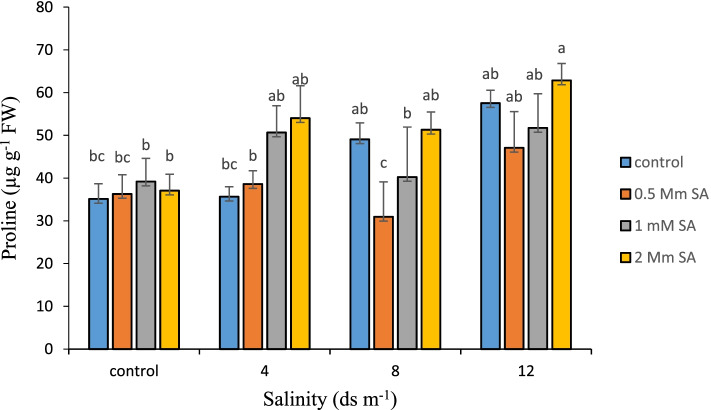
Comparison of the interaction of salinity and SA levels on the amount of proline in Damask rose (*p* ≤ 0.01). Different letters indicate significant differences between treatments by Tukey’s Test

By increasing salt in the Damask rose environment, the concentration of proline in the leaves also increased. The highest (62.83 μg g^− 1^ FW) level of proline was observed at the salinity level of 12 ds m^− 1^ and a concentration of 2 mM SA (Fig. 2) and the lowest occurred in the control treatment without salinity. Osmotic regulation is a very important stress defense mechanism, which is a type of adaptation to dehydration stress that, through the accumulation of soluble substances within cells, help cells to maintain their turgor pressure [63]. This regulation occurs through the production of organic matter such as proline, GB, proline betaine, and soluble sugars in roots and shoots [63]. Proline is the most important compound among osmotic protectors that play an important role in increasing resistance to salinity stresses to regulate osmosis or protect cell membranes [64]. Proline has been reported to play an important role in maintaining membrane stability by binding to membrane phospholipids, which changes the hydrated layer around biological macromolecules [65]. It has also been shown that following stresses, proline accumulates significantly in cells, which may be due to synthesis or reduction of its degradation [66, 67]. The use of SA increased the proline content and played a positive role in increasing plant resistance to salinity stress. SA has also probably produced a protective reaction by inducing the synthesis of intermediate compounds such as ABA, which induces proline production and reduces salinity damage in plants [18]. The concentrations of proteins, proline, and enzymes such as POD and APX in plants under high temperature stress with exogenous application of SA significantly increased but the activity of CAT decreased [19]. Tomato plants grown under normal conditions had a low proline content, but proline levels increased in both groups of plants under low water stress and plants treated with SA [9]. A report on the effect of SA on plants has shown that SA leaf spray increases the concentrations of carbohydrates, proteins, free amino acids, and proline [68, 69]. Our results are consistent with the above findings and show that with increasing salinity stress, the level of proline increases and the production of proline in plants can be increased by using SA.

### Malondialdehyde (MDA) content

**Fig. 3:**
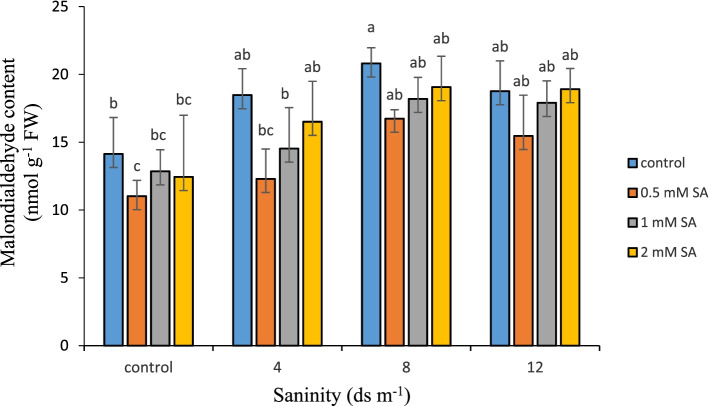
Comparison of the interaction of salinity and SA levels on MDA in Damask rose (*p* ≤ 0.01). Different letters indicate significant differences between treatments by Tukey’s Test

MDA forms as a result of degradation and oxidization of lipids which is known as an index for membrane damage. Therefore, when stress occurs, the amount of malondialdehyde increases in plants. According to the results of comparing the mean values (Fig. 3), plants under salinity stress (8 ds m^− 1^) that were not exposed to SA had the highest amount of MDA (20.8 nmol g^− 1^ FW); yet, in plants exposed to SA at 0.5 mM concentration with no stress applied, this value at its highest was found to be 11.02 nmol g^− 1^ FW. Accumulation of oxygen free radicals and the decrease in calcium concentration in cell walls under salinity stress leads to ion leakage and peroxidation of cell membrane fatty acids, which leads to the accumulation of MDA [70]. MDA accumulation is used as a biomarker to determine the extent of oxidative stress damage to cell membrane fatty acids and other biological molecules such as proteins, DNA and RNA [71]. The level of MDA accumulation varies depending on the type and severity of biological and abiotic stress [72]. SA treatment activates the antioxidant system, reduces free radicals, and protects membranes against lipid peroxidation [14]. With salinity plus SA treatment, the level of MDA reduced, which can be attributed to the ability of SA to inhibit the production of free radicals, because these free radicals lead to lipid peroxidation, they alter the production of cell macromolecules [73]. Salinity and drought can induce oxidative stress of ROS accumulation by increasing the level of MDA and other aldehydes. Another experiments showed that salinity alone increased MDA in plants, but when SA treatment was applied with it, MDA levels were reduced and the risk of oxidative stress reduced [46, 74, 75]. In this study, SA significantly reduced lipid peroxidation under salinity stress in Damask rose.

### Glycine betaine (GB) content

**Fig. 4:**
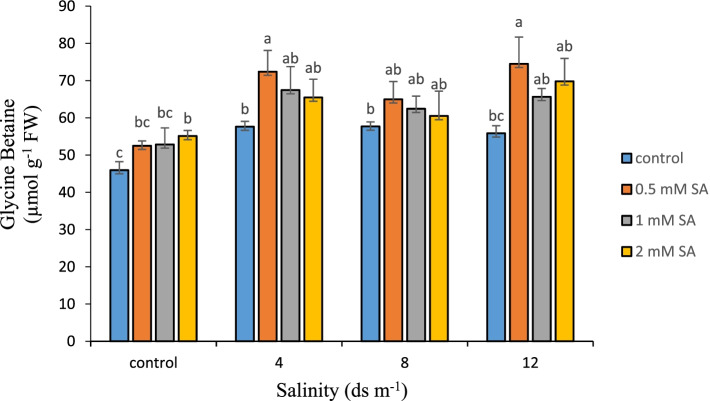
Comparison of the interaction of salinity and SA levels on Glycine Betaine in Damask rose (*p* ≤ 0.01). Different letters indicate significant differences between treatments by Tukey’s Test

Another non-enzymatic antioxidant mechanism in plants to deal with environmental stresses is the production of GB. This compound increases plant tolerance to stress by preventing lipid degradation and maintaining osmotic balance. The highest (74.51 μmol g^− 1^ FW) level of GB was detected in the salinity stress of 12 dS m^− 1^ with 0.5 mM SA. The lowest (45.96 μmol g^− 1^ FW) level of GB was found in the control treatment without salinity stress and without SA. According to Fig. 4, with increasing salinity level, the level of GB increased, which holds true when SA was used. GB is another compound that accumulates in plants in response to salinity stress and can be used as a cytoplasmic soluble substance to regulate osmosis like proline [66, 76]. Unlike proline, it is not metabolized rapidly after abiotic stress and can be an indicator of a previous stress on the plant [77]. Under stress, GB can protect photosynthetic activity by protecting photosynthetic carbon dioxide-stabilizing enzymes such as rubisco and rubisco activase, proteins, and lipids in thylakoid membranes [78]. Furthermore, GB directly protects the cell transcription machine from abiotic stresses and limits the overcharging of potassium ions generated by ROS by maintaining membrane cohesion through its role in reducing the production of ROS [79]. GB increases the expression of genes that scavenge ROS such as CAT and APX and reduces the accumulation of ROS. Numerous studies have shown that SA stimulates the production of GB [80]. The exogenous use of SA increases GB levels in abiotic stress [81]. The results of the present study also showed that with increasing the level of salinity stress, the level of GB in the samples increased and the application of SA increased the production of this substance in stressed plants, which is fully consistent with the above.

### Antioxidant capacity (AC)

**Fig. 5:**
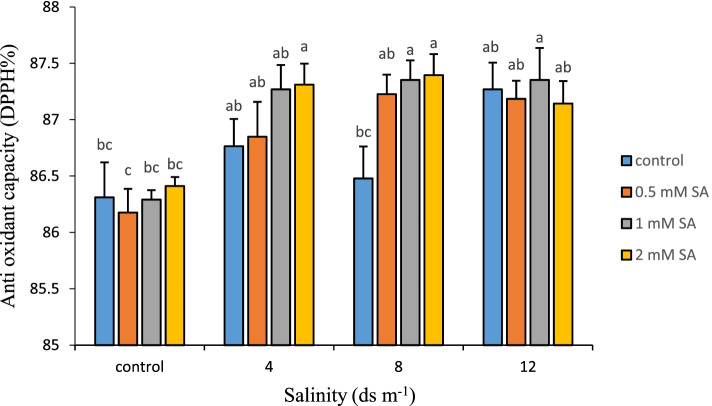
Comparison of the interaction of salinity and SA levels on antioxidant capacity in Damask rose (*p* ≤ 0.01). Different letters indicate significant differences between treatments by Tukey’s Test

AC is a system that eliminates ROS and traps them in plant cells. When stress occurs, plant cells act by activating antioxidant systems to reduce the adverse effects of stress. As it is clear in Fig. 5, with the increase of salinity in plants, the AC increased, and the use of SA also increased the AC. The highest (87.39%) ACs were recorded at 8 ds m^− 1^ salinity with 2 mM and then at a salinity stress of 12 ds m^− 1^ and 1 mM SA. The lowest AC was reported in the treatment without salinity stress with 0.5 mM SA. ROS are formed due to biological and non-biological stresses in plants. ROS are forms of atmospheric oxygen (O_2_) that have been partially reduced. When oxygen is excited, a unique oxygen (O_2_^•^) is formed. By transferring one, two, or three electrons to oxygen, superoxide (O_2_^−^), hydrogen peroxide (H_2_O_2_), or hydroxyl (OH^−^) radicals are formed, respectively. Unlike atmospheric oxygen, ROS have an unlimited ability to oxidize cellular biomolecules such as lipids, proteins, DNA, and RNA, which can lead to oxidative damage to cells [82]. When SA is used in the right concentration and time, it causes a temporary and transient oxidative stress in plant cells, which acts as a resilient process and increases the cell’s AC [8]. SA changes the activity of enzymes such as SOD, CAT, APX, or NAD (P) H oxidase attached to the cytoplasmic membrane of enzymes involved in the production or decomposition of H_2_O_2_, which leads to a temporary and slight increase in H_2_O_2_ content as secondary messenger to increase the AC of cells and induce other responses reducing the negative effects of stress. SA protects the plant from damage caused by oxidative reactions by increasing the activity of antioxidant enzymes as well as antioxidant mechanisms such as GB and proline. Foliar application of SA reduces the peroxidation of lipids and the amount of H_2_O_2_ by increasing the AC and further protects cell membranes and photosynthetic pigments and prevents chlorophyll catabolism [14]. The results also showed that the use of SA increased the production or activity of enzymes and antioxidant compounds in Damask rose, which are obvious by measuring the total AC.

### The activity of antioxidant enzymes (CAT, APX, GPX, POD, SOD)

The antioxidant enzymes such as CAT, SOD and peroxidase are regarded as one of the most important lines of defense in plants against biological stresses. Changes in these enzymes caused by salinity stress and salicylic treatment in *Rosa damascena* leaf samples are examined as follows.

**Fig. 6:**
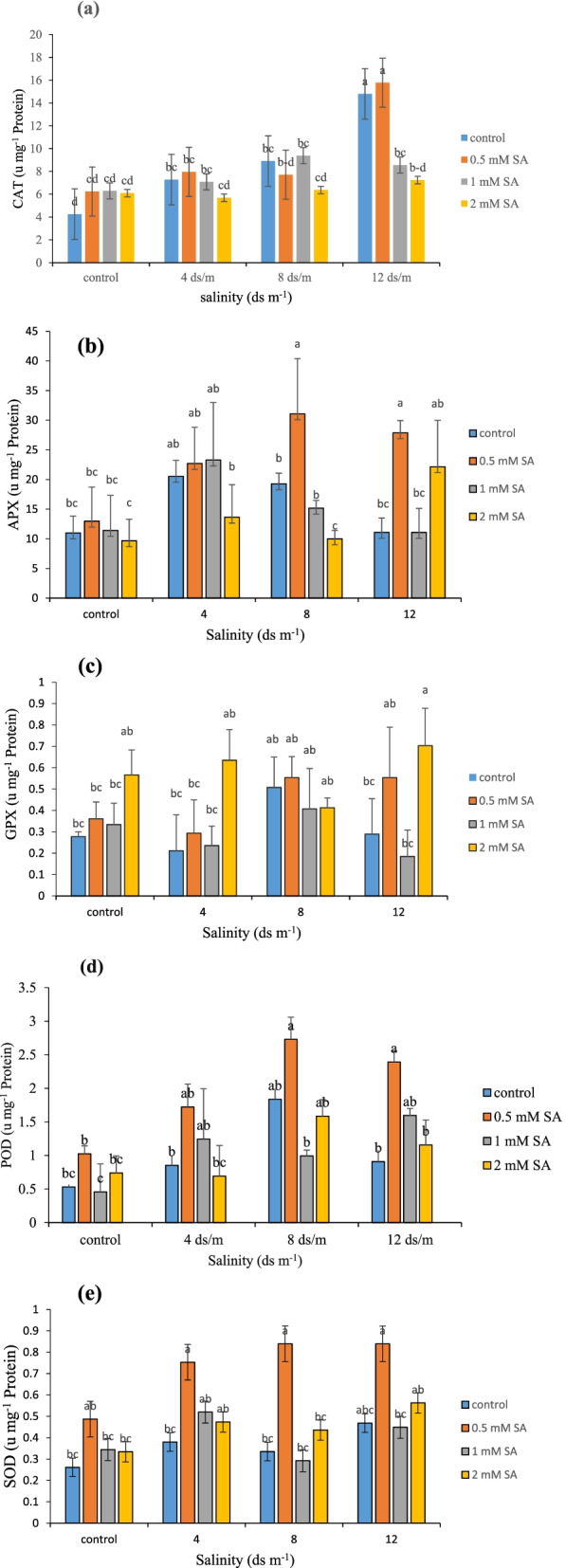
Comparison of the interaction of salinity and SA levels on CAT (**a**), APX (**b**), GPX (**c**), POD (**d**), and SOD (**e**) in Damask rose (*p* ≤ 0.01). Different letters indicate significant differences between treatments by Tukey’s Test

The CAT enzyme is one of the most prominent antioxidant systems in plants, when confronted with ROS caused by stress. Accordingly, regarding the plant examined in this research, the highest amount of catalase enzyme was found as 15.77 unit’s mg^− 1^ protein under level 12 ds m^− 1^ salinity stress and the application of SA at 0.5 mM concentration; nonetheless, the value reduced by 73.05% in the control treatment (involving no salinity stress and no SA application) (Fig. 6a). The production of oxygen free radicals is induced in response to various abiotic stresses such as salinity, drought, and high temperatures [83]. Increased [9] and/or decreased [17] activity of CAT under drought stress has been reported. Decreasing the activity of CAT enzyme may be related to its optical inactivation and inhibition of enzyme re-synthesis in the dark, which causes the accumulation of hydrogen peroxide and damage to cell membranes. Furthermore, an increase or decrease in CAT activity has been reported after the use of growth regulators such as SA, which seems to depend on the concentration and method used (soaking, foliar spraying, solubilization, injection, etc) as well as the condition of the plant, including the developmental stage, the oxidative balance of the cell and previous adaptations by biotic and abiotic stresses [84]. It has been reported that SA treatment leads to a temporary decrease in CAT activity and an increase in hydrogen peroxide levels, which plays a key role in establishing systemic acquired resistance [17]. It has been shown that when the concentration of internal SA exceeds a certain level, they bind directly to CAT enzymes and inhibit their activity [46]. The exogenous application of SA increases antioxidant enzymes such as SOD, POX and CAT to combat stress [9, 85]. The results of the present study also showed that different concentrations of SA may have contradictory effects and depend on the concentration used. In this study, the optimal SA concentration of 0.5 mM was considered, but higher concentrations had an adverse effect on CAT activity.

The results of mean value comparison shown in Fig. 6b demonstrated increased APX enzyme activity due to increased salinity stress. Results also showed the lowest amount of this enzyme as 9.66 units mg^− 1^ protein under no salinity stress and the application of SA at a concentration of 2 mM; increasing the level of salinity stress to 8 ds m^− 1^ and applying SA at 0.5 mM concentration increased APX enzyme activity by 68.9%. SOD, CAT, POD, APX, and GR are important enzymes in the antioxidant defense system of plants. Although SOD acts as the first defense against ROS, its end product (hydrogen peroxide) is toxic to the cell and must be removed from the cell by CAT or APX of the glutathione ascorbate cycle [86]. A balance is established between SOD, CAT, and APX to determine the levels of superoxide radicals and hydrogen peroxide. Numerous reports indicate that the removal of hydrogen peroxide is achieved by APX and CAT [87, 88]. It also increased the accumulation of antioxidant enzymes such as SOD, CAT, GR, APX and reduced oxidative damage and increased Fv / FM ratio under stress [5]. In this study, the use of SA increased the activity of APX and the peak activity occurred at a concentration of 0.5 mM.

GPX is another antioxidant enzyme in plants which acts in the second line of defense and transforms the hydrogen peroxide produced by these enzymes into water and oxygen. Salinity stress at level 12 ds m^− 1^ significantly affected the activity of this enzyme (Fig. 6c), which showed the lowest (0.18 units mg^− 1^ protein) and highest extent of activity (0.7 units mg^− 1^ protein) when exposed to SA at concentrations of 1 and 2 mM, respectively. A study on “Rockfire” roses found that increasing the level of ROS in the leaves of plants grown in vitro under salt stress increased the activities of CAT, SOD, GPX and APX [89]. In another study, activities of CAT, SOD and GPX increased in salinity stressed Damask roses [90]. Foliar application of SA in plants exposed to salinity reduces the accumulation of sodium and increase absorption of potassium and magnesium. It also increase the activities of GPX and CAT [91]. Use of SA also increased the expression and production of GPX [91]. The results of this study are consistent with earlier reports, and the results show that the use of SA increases the production of GPX.

The POD enzyme is also another important factor that removes ROS under stress and plays a significant role in reducing the adverse effects of stress on plant cells. When exposed to salinity stress at level 8 ds m^− 1^ and SA at 0.5 mM concentration, the POD enzyme activity in the Damask rose significantly increased by 83.45% compared to plants that received SA at a concentration of 1 mM, with no exposure to stress (Fig. 6d). Consequently, these results show that the POD enzyme activity raises at higher levels of salinity stress. POD breaks down hydrogen peroxide by using phenolic substances as electron donors [92]. The combined action of POD and CAT is required to protect plant cells from hydrogen peroxide produced by SOD. Increased POD activity due to salt stress has already been reported [63] Exogenous application of SA to tomato plants under drought conditions increased the activity of antioxidant enzymes, including CAT, POD, and SOD, as well as proline content [9]. As a growth regulator, SA plays an important role in cell growth, respiration, stomatal closure, senescence, increasing POD activity and photosynthesis under stress in plants [93]. In this study, the use of different concentrations of SA increased POD activity in Damask rose samples, which is consistent with the above results.

The results of mean value comparison regarding the SOD enzyme activity also showed that increased levels of salinity stress increases the activity of this enzyme (Fig. 6e). Accordingly, 68.67% increase in the activity of this enzyme was observed under salinity treatments of 8 and 12 ds m^− 1^ compared to the control treatment. Abiotic stress such as salinity stress induces the production and accumulation of reactive oxygen species, which are harmful to cells at high concentrations. The production of these compounds induces lipid peroxidation, inactivation of enzymes, destruction of nucleic acids, and destruction of cell membranes [94]. SOD is the first and most important enzyme in the detoxification process of ROS compounds, which plays a vital role in cell defense mechanisms against the risk of hydroxy radical formation (OH) by converting superoxide radicals to H_2_O_2_. The resulting hydrogen peroxide is then purified by CAT and APX. Because SOD inhibits superoxide radicals, it is the first antioxidant enzyme to counteract the destructive effects of reactive oxygen species. Farooq et al. (2010) [95] investigated the effects of SA on plants under drought stress and showed maximum activity of SOD was at 1 mM of SA. The results of this study are consistent with the above findings and show that the administration of SA increases SOD activity in Damask rose, and it was also found that different concentrations of SA could lead to different results.

### Relative expression of CAT gene

**Fig. 7:**
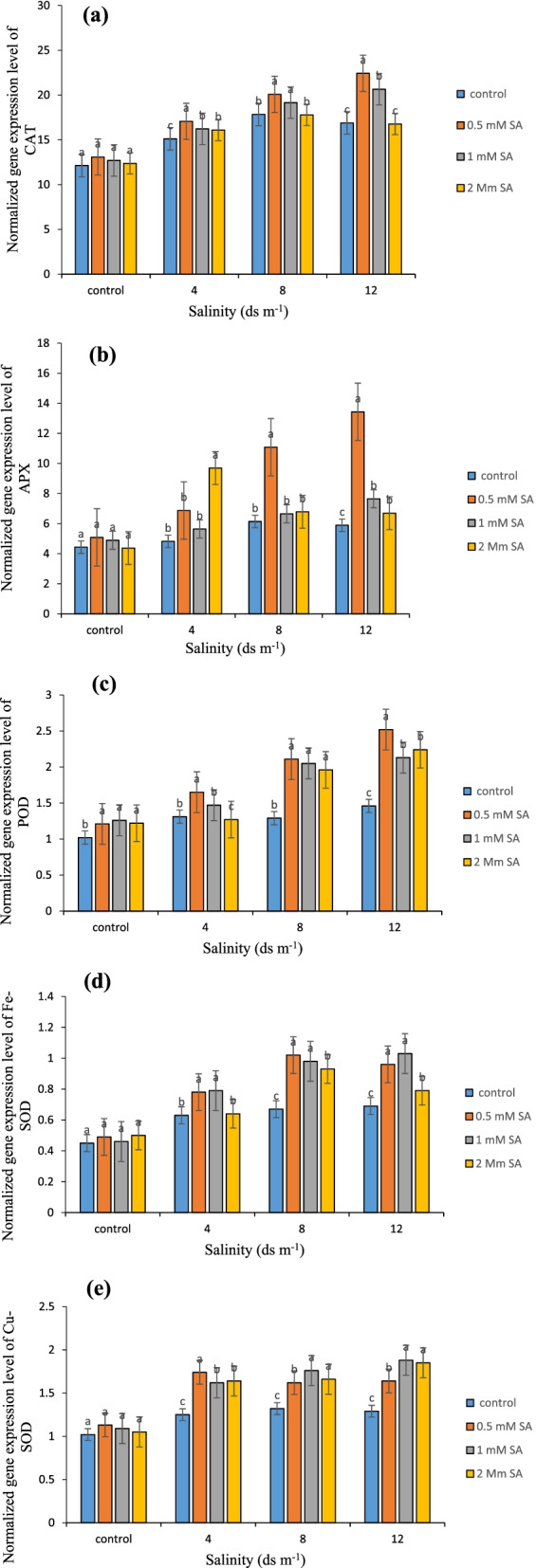
Relative expression of CAT gene (**a**), APX gene (**b**), POD gene (**c**), Fe-SOD gene (**d**), and Cu-SOD gene (**e**) in Damask rose under salinity stress and SA treatment. (*p* ≤ 0.01). Different letters indicate significant differences between treatments by Tukey’s Test

CAT is an antioxidant enzyme functioning at the forefront of the plant’s defense system during oxidative stress. The expression of the gene related to this enzyme indicated the activity of this gene in the plant leaves. Figure 7a shows that in treatment without salinity stress, there was no significant difference between different levels of SA in terms of CAT gene expression. Thus, at salinity stress of 4 ds m^− 1^, the highest level of gene expression was observed in 0.5 mM SA (17.7) and the lowest expression was in the treatment without SA (15.12). At the salinity levels of 12 ds m^− 1^, the highest relative expression of the CAT gene was observed in 0.5 mM SA (22.43), and the lowest expression was observed in the treatment without SA and 2 mM SA (Fig. 7a). With the increase of salinity stress, the expression level of CAT gene also increased, and the use of SA increased the expression of this gene at different levels of salinity stress. The optimal concentration of SA was determined to be 0.5 mM. Studies on the expression of CAT gene in *Cynodon dactylon* have shown that the expression of this gene in young leaves of a tolerant cultivar under 400 mM salinity stress was higher than a susceptible cultivar [96]. Similarly, it was shown that the increase in CAT and APX gene expression in rapeseed under drought stress was higher in a tolerant cultivar than in a susceptible cultivar [81]. The use of SA increased the expression of the CAT gene under salinity stress [79].

### Relative expression of APX gene

APX is also one of the key antioxidant enzymes in the plant immune system. This enzyme is considered as the main factor in the reduction of free radicals such as H_2_O_2_, which can minimize the damage caused by oxidative stress [97]. Therefore, the higher expression of this gene indicates the high activity of this enzyme in the plant. Results concerning the comparison between mean values (Fig. 7b) with respect to the relative APX gene expression demonstrate increased expression of this gene (13.43) at higher levels of salinity stress (12 ds m^− 1^) and treatment with SA at 0.5 mM concentration. In addition, in case of applying no salinity stress and using SA at a concentration of 2 mM, the relative APX gene expression would be 4.37 at its lowest. Therefore, it can be concluded that the APX activity increases when the level of salinity stress is raised. The expression of the APX gene in tolerant cultivars was much higher than in sensitive cultivars under abiotic stress [98]. The APX gene expression increased under salinity stress in three melon ecotypes [32]. In tomatoes [32] and strawberries, increased levels of APX gene expression were also reported when plants were exposed to stress [63]. In a study, the use of SA in plants exposed to cold stress increased the expression of the APX gene [99, 100]. Our study also showed that the application of SA in salinity stress of Damask rose plants increased the expression level of the APX gene, which in turn increased the tolerance of plants to salinity stress.

### Relative expression of POD gene

POD activity can be easily detected throughout the life of various plants from early germination to aging by controlling cell elongation, defense mechanisms, and several other functions. PODs are involved in many cellular processes such as auxin metabolism, wood formation, cross-linking in the plant cell wall, response to environmental stresses [101], phenol oxidation and resistance to salinity stress [102]. Phenolic compounds in leaves are oxidized to quinones by POD and primarily by polyphenol oxidase, which is used under oxidative stress as a reactive agent against the production of ROS [84]. Regarding relative expression of the POD gene, Fig. 7c shows that there was no significant difference between different concentrations of SA in the control treatment. At salinity stress of 4 ds m^− 1^, the highest and lowest expression levels were reported in 0.5 mM and 2 mM SA, respectively. At 8 and 12 ds m^− 1^ of salinity, the highest relative expression level was in the concentration of 0.5 mM SA and the lowest was in the treatment without SA. The use of SA increased the expression level of the POD gene. The results of gene expression are consistent with the results obtained from the activity of this enzyme, which shows that increasing the gene expression of this enzyme has a direct role in its activity. The POD gene expression has been reported in salinity stress of various plants [103]. There are many studies showing that SA induces POD gene expression and activity in stressed plants [12, 104]. The study was found that the use of anti-stress compounds such as SA and jasmonic acid increased the expression level of POD gene and increased the plant’s ability to cope with oxidative stress [105]. The results of this study are in line with the above findings and showed that the use of SA during salinity stress in plants increased the expression of POD gene in Damask rose plants.

### Relative expression of SOD (Fe-SOD and cu-SOD) gene

SOD enzyme is one of the most important plant antioxidants acting against environmental stresses. This enzyme converts the ROS to H_2_O_2_, which is then converted to water and oxygen by other enzymes. The enzyme SOD is divided into different types according to the co-factor it has. In this study, iron and copper-based oxides were evaluated. The results of relative gene expression regarding Fe-SOD showed that the highest extent of expression for this gene was achieved as 1.03 and 1.02 under salinity stresses of 12 and 8ds m^− 1^, and the application of SA at 1 and 0.5 mM concentration, respectively (Fig. 7d). Additionally, in case of no salinity stress and SA concentration of 1 mM, the lowest expression would be observed with a value of 0.46. The highest amount of relative Cu-SOD gene expression (1.88) was found under the highest salinity stress (12 ds m^− 1^) where no significant differences were identified between treatments involving SA at concentrations of 1 and 2 mM (Fig. 7e). In addition, reducing the salinity stress to 0 (control) decreased the amount of expression in this gene; the lowest amount of expression (1.02) was found after using SA at a concentration of 0 (control). SODs are metal enzymes that catalyze the conversion of superoxide radicals to oxygen and hydrogen peroxide and are at the first line of defense system against oxidative damage. There are three main forms of these enzymes in plants. The metals used in their structure are classified into Cu/Zn-SOD, Mn-SOD, and Fe-SOD. The loci of activity of these isoforms also differ: Mn-SOD in mitochondria, Cu / Zn-SOD in cytosol and chloroplasts, and Fe-SOD in chloroplasts [106]. As mentioned in the results section, the expression of Fe-SOD and Cu-SOD genes in leaves gradually increased with increasing salinity treatment and reached its highest level at the highest stress level (12 dSm^− 1^). These results are consistent with the results obtained from the study of the effect of salinity stress on the shoot of peppermint in which Fe-SOD gene expression began with salinity stress and with increasing stress, gene expression increased significantly [107]. In the study of the effect of salinity on plants, salinity stress significantly increased the activity of total SOD and Fe-SOD isozymes compared to the control [108]. In an experiment, SOD activity increased under sodium chloride stress in the roots and shoots of barley [105]. In a study on soybeans, the expression of the Cu/Zn-SOD coding gene was induced under cadmium stress [109]. SA increased the level of SOD gene expression and consequently the activity of SOD [8]. Exogenous use of SA increased the expression level of the SOD gene, thereby increasing the production of related enzymes [110–113]. This study also showed that foliar application of SA on the leaves of Damask rose increased the expression level of the SOD gene, which was consistent with the above.

## Conclusion

Nowadays, salinity stress is considered one of the most significant abiotic stresses as it damages a large number of agricultural products and reduces their performance. As shown in this study, increasing the concentration of salt reduces the growth speed which points to reduction in dry and fresh weight of leaves, aerial organ and root in the Damask rose. Additionally, increasing the level of salinity stress up to 12 ds m^− 1^ affected the amount of chlorophyll, root length and leaf total area, all of which reduced significantly compared to plants under no stress. However, many studies have highlighted the application of compounds that reduce the negative effects of stress and increase plant resistance and tolerance against stresses. Consequently, in this study, the effect of SA was examined as a plant intra- and extracellular regulator that is known to be effective in increasing plant resistance against salinity stress. Even at low concentrations (0.5 mM), SA managed to neutralize the negative effects of salinity; therefore, it was identified in this research as a suitable regulator for *Rosa damascena*. Under salinity stress, the enzyme antioxidant defensive systems and the genes involved were activated in *Rosa damascena*; accordingly, the presence of SA increased the enzyme and antioxidant gene expressions in the plant. These gene-related mechanisms were able to trap ROS which, in turn, raised plant resistance against salinity. As a result, considering the positive effect of SA on plant growth during stress, it can be regarded as a suitable regulator for the Damask rose, even at low concentrations. Nonetheless, it is recommended to examine other regulators such as Humic acid, Polyamines, Amino acids and etc. at different concentrations to acquire more robust results.

## Data Availability

All generated or analyzed data were included in this article. The raw datasets obtained during the current study are available from the corresponding author on reasonable request.
